# Supplementary material for: Patterns of Intron Gain and Loss in Fungi
Source: PLoS Biol. 2004 Nov 30;2(12):e422. doi: 10.1371/journal.pbio.0020422 (PMC532390; doi:10.1371/journal.pbio.0020422)
Supplement: Table S1 — Also available at http://genes.mit.edu/NielsenEtAl/. (4.3 MB ZIP). [file pbio.0020422.st001.zip › NielsenEtAl/html/93.html]

AN6067.1.NCU00366.1.MG08866.1.FG05425.1


```
 CLUSTAL W (1.82) Multiple Sequence Alignments - Introns Inserted


Sequence 1: NCU00366.1	425 aa
Sequence 2: MG08866.1	430 aa
Sequence 3: FG05425.1	420 aa
Sequence 4: AN6067.1	423 aa
Alignment Length: 439 aa
Number Identitical Residues: 202 aa
Alignment Score (without introns) 9962


MG08866.1 	--MSLINVRRDNKDVFYRYKMERLQTKIEGKGNGIKTVVVNLTSVAQSLARPGAYLIKYF
NCU00366.1	-MGALINVRRDNPDPFYRYKMERIQTKIEGKGNGIKTVVVNLSSVAQSLARPGGYLIKYF
FG05425.1 	--MALVNVRRDVSDAFYRYKMERLQTKIEGKGNGIKTVVVNLSSVAASLARPGSYVIKYF
AN6067.1  	MAPNNINIRRDVSDPFYRYKMEPLQSKIEGKGNGIKTVIVNLNSVAGSLSRPPAYVIKYF
          	     :*:***  * ******* :*:************:***.*** **:** .*:****

MG08866.1 	GFELGAQTNIDPADDRWIINGAHDAAKLQDYLDGFINKFVLCKKCKNPETDVIIKDGNIS
NCU00366.1	GFELGAQTNIDPPDDRWIINGSHEAAKLQDLLDGFIAKFVLCKKCKNPETVVQIKDEKIT
FG05425.1 	GFELGAQTNIDPKDDRWIINGAHDAPKLQDHLDGFINKFVLCKKCKNPETDVVIKDDHIT
AN6067.1  	GFELGAQANAKPTDDRWIINGAHDSRKLQDYLDGFITKFVLCKKCKNPETDVIIKDEKII
          	*******:* .* ********:*:: **** ***** ************* * *** :* 

MG08866.1 	LDCKACGARSDVDLRLKLSGFILKN-VPKKTKKDKAERKAARKAKQNGTAKDGEGSGEE-
NCU00366.1	LDCKACGQRSKVEPQLKLTSFILKN-VPKKSKKDKAERKAARKARQNGGDK--EGSGDE-
FG05425.1 	LDCKACGKISDVDLRLKLSGFILKN-QPKKGKKDKAERRAAKKARQNGNAANGTNGGSD-
AN6067.1  	LDCKACGQRSDVDSRLKLSTFILRNNTSGKGKKDKSTKKTRRERNKEKEAANGENNGSPG
          	*******  *.*: :***: ***:*. . * ****: ::: :: .::    .. ..*. .

MG08866.1 	HSPDGSGKENGDKNGHNGADDGSDDDDALTRKIKNEAKEISSNGGGKADDDEWAVDMSAE
NCU00366.1	NGSDQASPAIEDKNIEYASDD-----DELTRKIKSEAQSLQLGKREAPKEEEWAVDMSEE
FG05425.1 	EASDNGSNENADED--AGSDD-------EFQKVQAEAAAAAQEN--EVKEHEWAVDMSEE
AN6067.1  	ESNSDNGDENEDGALEAGSDD------ELTRRINTAAQGIEAED--EIEDDNWNVDVSEE
          	.. .  .    *     .:**         ::::  *           .:.:* **:* *

MG08866.1 	AVKARQQSLPGEFKQKLNINGEDEDEDGEGGNSVYDQLGTWILDQAAEKGNIDKVDDIAI
NCU00366.1	AVRARQQNLPGEFKQKLVLNGE-EDEEEEGGNTVYDQLGTWIEDEAKAKGGINNVDDVEI
FG05425.1 	AVKARQASLPGEFKAKLNIGDD-DDEDGEGGPTIYDELGDWIQSQAEEKGGIDKVESIDI
AN6067.1  	AVKARAKELPDDLKRALALDEG--DDEGADGPTAYDELGSWVLDTATEKGGISKVEDVEI
          	**:**  .**.::*  * :.    *::  .* : **:** *: . *  **.*.:*:.: *

MG08866.1 	YVKAKEFGIETKHRTVLVLVQTLFNENICAQITKRAGMLKQ0IVTSERHEKALLGGTERL
NCU00366.1	YLKAKELGIDAKHRTVQVLVQCLFDENIVAQIPKRAPMLKR~LVTSERHEKALLGGTERL
FG05425.1 	YVKAKELGIEGKHRTVLVLVQTIFDKNIVAQISKRASMLKQ0FVTSERHERALLGGTERL
AN6067.1  	YLKAKELGIETKHKTLAVLAQTIFDEKIAKQVDGRAPLLKK~MITSERHEKAFLGGTERF
          	*:****:**: **:*: **.* :*:::*  *:  ** :**: ::******:*:******:

MG08866.1 	IGQLSKDKPEMFDKIVKILQLYYHHDLISEEVVTKWGTHASKKYVDLSTSKKVRKSAEPF
NCU00366.1	LADLGK---ESYDKIVKILQLYYHHDLCSEEVITKWGSKASKKYVDGSTSKKIRKAAEPF
FG05425.1 	VGGLGADHPEMFQSIVKILQLYYHHDLISEEVVTKWGSKASKKYTDISTSKKVRKAAEPF
AN6067.1  	LG---KEHPELISQVPAVLLGYYQNDLVSEETLKAWCSKASKKYVDIQTSRKVRKAAEPF
          	:.    . .*  ..:  :*  **::** ***.:. * ::*****.* .**:*:**:****

MG08866.1 	LNWLAEAE--EEDSDDE---
NCU00366.1	LTWLAEAESEEEESEEEDDE
FG05425.1 	LTWLAEADSEEESSDEE---
AN6067.1  	LQWLESAESEEESEDDE---
          	* ** .*::.**..::*
```
